# Building on and tailoring to: Adapting a cancer caregiver psychoeducational intervention for rural settings

**DOI:** 10.1002/cam4.70187

**Published:** 2024-09-05

**Authors:** Erin E. Kent, Kelly R. Tan, Zev M. Nakamura, Jesse Kovacs, Mindy Gellin, Allison Deal, Eliza M. Park, Maija Reblin

**Affiliations:** ^1^ Department of Health Policy and Management, Gillings School of Global Public Health University of North Carolina at Chapel Hill Chapel Hill North Carolina USA; ^2^ Lineberger Comprehensive Cancer Center University of North Carolina at Chapel Hill Chapel Hill North Carolina USA; ^3^ Cecil G. Sheps Health Services Research Center University of North Carolina at Chapel Hill Chapel Hill North Carolina USA; ^4^ Department of Health and Community Systems University of Pittsburgh School of Nursing Pittsburgh Pennsylvania USA; ^5^ Department of Psychiatry University of North Carolina at Chapel Hill Chapel Hill North Carolina USA; ^6^ Department of Family Medicine, Larner College of Medicine University of Vermont Burlington Vermont USA

**Keywords:** caregiver, dissemination, implementation science, intervention, psycho‐oncology, psychosocial, rural, social support

## Abstract

**Introduction:**

Rural cancer caregivers experience obstacles in accessing services, obtaining respite, and ensuring their care recipients receive quality care. These challenges warrant opportunities to participate in evidence‐based behavioral intervention trials to fill support gaps. Adaptation to rural settings can facilitate appropriate fit, given higher caregiver service needs and unique challenges. We present findings from the adaptation process of a psychoeducational intervention designed to support cancer caregivers in rural settings.

**Methods:**

We adapted Reblin's CARING intervention, designed for neuro‐oncology, to target caregivers of rural cancer patients across cancer sites. First, we conducted formative work to determine the unmet social and supportive care needs rural cancer caregivers faced. We used the Framework for Reporting Adaptations and Modifications to Evidence‐based Implementation Strategies (FRAME‐IS) to guide the modifications. To conduct the adaptation, we elicited feedback through qualitative interviews of seven caregivers and three cancer hospital staff and thematic analysis to inform intervention modifications. Our qualitative study was guided by the Consolidated Criteria for Reporting Qualitative Research (COREQ).

**Results:**

Interviews revealed that service access was a pressing need, along with financial (e.g., treatment costs, employment challenges) and geographic barriers (e.g., distance to treatment, road conditions). We modified content, training, and context using the FRAME‐IS steps. Changes enhanced fit through the following adaptations: changes to social support domains, session content, interventionist training, resource offerings, screening and recruitment processes, and virtual delivery.

**Discussion:**

Challenges to establishing successful psychosocial oncology interventions may be improved through participant‐centered approaches and implementation science. Additional systemic challenges, including lack of systematic documentation of caregivers, persist and may especially disadvantage under‐represented and underserved groups, such as rural dwellers. The enCompass intervention is undergoing ongoing single‐arm pilot of rural cancer patient/caregiver dyads targeting caregiver coping self‐efficacy and patient/caregiver distress (Clinical Trials #NCT05828927).

## Graphical abstract

Challenges to establishing successful behavioral interventions in psycho‐oncology may be improved by using participant‐centered approaches and incorporating implementation science frameworks. We detail the creation of enCompass Carolina, a supportive care intervention for caregivers adapted from an existing program designed for the neuro‐oncology setting, for use across multiple cancer sites and in the rural setting.

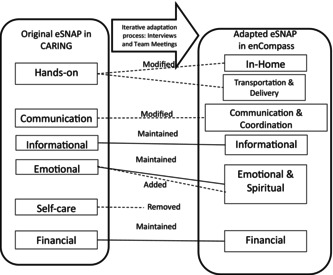

## 1 BACKGROUND

Family caregivers fulfill increasingly important roles in cancer care delivery, offering essential social support to cancer patients and survivors, often to the detriment of their own physical and psychosocial health. Caregivers of rural cancer patients have been deemed a priority underserved population by the National Cancer Institute given many unmet needs and cancer health disparities as compared to urban populations.^1^ Despite a growing number of evidence‐based interventions and programs to improve caregiver well‐being through psychoeducation, skills training, and emotional support,^2^, ^3^, ^4^, ^5^ few are offered in a widespread or systematic manner.^6^ Existing supportive interventions for caregivers are not always accessible to rural residents given geographic constraints and cost, and those that may be available suffer insufficient funding and inadequate caregiver identification and assessment pathways.^3^, ^7^

Furthermore, cancer centers with smaller patient volumes are less likely to offer caregiver support programs,^8^ many caregivers are not eligible for existing caregiver support programs,^9^ and certain aspects of cancer caregiving warrant specific tailoring, for example: often rapid patient health decline and the need for complicated, intensive, and costly treatment regimens. Although there are a small number of efficacious supportive interventions for rural caregivers of persons with dementia,^10^ very few exist for rural cancer caregivers.^11^ Thus, there is continued need to adapt and tailor caregiver interventions to populations with unmet needs though cancer care delivery.

Researchers are encouraged to focus on innovation, and as such, are often incentivized to build new interventions. With the rise of the field of implementation science, this paradigm is shifting. However, there are few examples of successful adaptation of caregiver interventions across sites and study populations. A recent systematic review indicates that many manuscripts on caregiver interventions lack key details about study design that would lead to broader implementation, including acceptability from caregivers, adoption practices, replicability, and resources needed to execute the intervention.^3^ As such, there is a need to create a roadmap to help research teams shift their collaborative focus from intervention development to adaptation and expansion of existing interventions to new contexts.

In this paper, we outline how our team adapted CARING, an intervention designed to support caregivers of neuro‐oncology patients, into enCompass, designed for caregivers of rural patients with any type of cancer. Based on social cognitive theory^12^ and Fletcher's adaptation of stress and coping models for caregivers of cancer patients,^13^ the CARING intervention targets appraisal by helping caregivers identify supportive assets among their existing social networks, stress responses by building support‐seeking and problem‐solving skills, and stressors by providing supportive care resource information.^14^, ^15^, ^16^ We build on the strengths of this intervention by adapting, rather than reinventing, the intervention for caregivers of cancer patients in rural settings.

## 2 METHODS

We used the Framework for Reporting Adaptations and Modifications to Evidence‐based Implementation Strategies (FRAME‐IS) as a model to guide our reporting of the key outcomes of this process. The FRAME‐IS provides guidance on tracking modifications to implementation strategies, with the following stages: (1) defining what is being modified (content, evaluation, training, context), (2) identifying the nature of the modification (tailoring, removing, adding, etc.), (3) articulating the modification goal (increasing reach, adoption, acceptability, appropriateness, etc.), and (4) identifying the rationale for modification (e.g., available staffing, recipient needs). We describe our process as well as key factors necessary for success and lessons learned. The adaptation process of the CARING intervention into enCompass was iterative. We first describe the original intervention and the steps undertaken for its modification.

### 2.1 The CARING intervention

The caregiver intervention that we adapted, CARING, pairs a web‐based social network visualization tool and resource list (the electronic social network assessment program; eSNAP) with personalized caregiver navigation, consisting of 8 weekly manualized phone sessions with a caregiver navigator to address caregiver social support and coping needs.^14^, ^17^, ^18^ The intervention was developed for neuro‐oncology outpatient populations with extensive pilot work and input from caregivers, as well as other stakeholders, including clinicians and experts in user‐centered design and navigation programs.^16^, ^18^ The intervention has been implemented in a primarily urban National Cancer Institute‐designated Comprehensive Cancer Center.

The CARING intervention provides a flexible structure for caregivers to reflect on their own unique support resources and needs and scaffolding to focus on developing or refreshing coping skills. Within this structure, the content can be tailored to each individual caregiver. For example, navigation sessions focus on coping skills (e.g., asking for help, setting boundaries) and navigators use problem‐solving and motivational interviewing to help caregivers interpret what this means in their own situation, practice relevant skills that may be useful in achieving caregiver‐directed goals, and identify informal social support (i.e., friends/family) or formal services that can support them towards their goals and as a caregiver more broadly.^18^ Our determination that CARING was an appropriate fit for both a rural and a more general cancer caregiving population was largely based on the flexibility of the intervention to be tailored to individual caregiver circumstances and existing research suggesting that rural caregivers have unique social contexts and needs that match the CARING framework.

### 2.2 The new target population: Caregivers of rural cancer patients

Caregivers of rural patients have both shared and unique needs with other caregiver populations. Rural residents are more likely to be caregivers than non‐rural residents, are more likely to provide more hours of care (>20 h per week),^19^ and were more likely to report poor‐to‐fair health than non‐rural caregivers,^20^ demonstrating high need. In general, rural as compared to urban residents also reported more reliance on peer support and informal networks,^21^ although non‐Hispanic Black adults residing in rural areas report higher loneliness^22^ and rural residents may demonstrate more reluctance to ask for help. However, rural caregivers, especially those who are Black, commonly report needing help with accessing services.^23^

### 2.3 Iterative approach to intervention adaptation

First, to confirm the fit of CARING for adaptation to rural cancer caregivers and identify potential components and characteristics to modify, we explored the research literature. Second, we conducted a formative qualitative study guided by Consolidated Criteria for Reporting Qualitative Research (COREQ) guidelines and consisting of 41 semi‐structured interviews with 24 rural caregivers and 17 hospital staff, including physicians, nurses, social workers, chaplains, healthcare administrators, and cancer care navigators. Findings from this formative study published elsewhere.^24^, ^25^ Briefly, the qualitative interview data were coded following COREQ guidelines, analyzed thematically, and findings discussed among the study team for relevance to our intervention adaptation, which included experts in caregiving, care delivery, oncology, nursing, and behavioral science. Based on our literature review and this formative study, several preliminary modifications of the CARING study were identified, largely focused on terminology and changes to the domain structure of the social support categories. As part of the formative process, we also identified formal cancer and rural caregiving resources, which were vetted by clinical social workers at the cancer hospital and our study team.

Next, guided by the FRAME‐IS model and with funding obtained from The Duke Foundation, we began our direct adaptation by conducting a new set of qualitative interviews with seven rural cancer caregivers and three cancer center clinicians to gather feedback on the intervention components: the CARING study eSNAP web‐based tool and the 8‐week navigation sessions. The eSNAP tool is an interactive, personally tailored program that stores user information in a secure, cloud‐based server that users can return to at any time with a log‐in and password. The tool leads users through a series of screens that describe different dimensions of social support and first asks participants to enter the names of people that can currently provide that support, as well as indicate if they are a family member, friend, or another relationship. After the social support prompts, the user can then choose to add selected resources identified by the research team as being possible fits for providing relevant support. The tool then demonstrates two sets of supports, with one display being “people” and another display being “resources” that can serve as visual reminders of support for users to refer back to, print (or the study team can print for them). Additional information about the original eSNAP tool is previously published.^14^, ^16^

We identified potential qualitative interview participants by screening electronic health records at the cancer hospital for patients diagnosed with stage II or III cancer, undergoing active treatment, and who resided in a rural zip code.^26^ We introduced the study to eligible patients and asked them if they had a caregiver, if that caregiver was available. If so, we approached the caregiver to introduce the study to them. We also interviewed clinical staff that had encounters with caregivers, including one oncology social worker and two staff members of the hospital patient navigation team. Each participant was introduced to eSNAP, the caregiver workbook, and eight sessions of intervention content. We conducted virtual interviews with each participant with a focus on acceptability and potential barriers.

### 2.4 Analytical methods

Qualitative interview data were analyzed thematically to evaluate potential modifications or adaptations. Transcripts generated from the interviews were entered into Dedoose qualitative analysis software for coding and synthesis, coded by two independent coders on the study team, and discussed by the study team to resolve discrepancies and build consensus around themes. Thematic content analysis was applied to the transcripts under COREQ guidance.^27^ Throughout the iterative adaptation process, our team met frequently with the original developer and study staff of the CARING intervention, and potential changes to components of the intervention were discussed and documented. All research procedures were conducted in compliance with human subjects, and informed consent was obtained. Ethical approval was provided by the University of North Carolina at Chapel Hill Institutional Review Board (UNC IRB# 22‐1840).

## 3 RESULTS

FRAME‐IS stages, including modification target; nature of the modification, modification goal, and rationale for modification, are used to describe our results across iterative stages of our adaptation process, separated into content, training, and context domains below. Modification exemplars in the adaptation of the CARING study for enCompass are shown in Table 1.

**TABLE 1:** Components of the CARING Study modified for enCompass Carolina according to FRAME‐IS domains.

What is being modified	Nature of the modification	Modification goal	Data source for modification
Content			
eSNAP support domains	Refining terminology (“Hands‐on” to “in‐home”) Refining emotional to include spiritual; refining communication to include coordination Teasing out transportation and delivery from hands‐on Eliminating explicit self‐care	Increase acceptability and appropriateness (participant level)	Caregiver interviews; literature on rural caregivers
Session content	Emotional words at check‐in and at conclusion of sessions	Increase acceptability (institution/clinic level)	Caregiver, clinician interviews
Training			
Navigator title	Change title to “coach”	Increase acceptability (institution/clinic level)	Caregiver, clinician interviews
Navigator training	Tailoring to current population and simplification	Increase appropriateness and feasibility (intervention level)	Staff discussions
Context			
Personnel	Replacing professional lay navigators with student interns	Decrease cost; increase sustainability (intervention level)	Staff discussions
Population/enrollment criteria	Eliminating patient participation requirement	Increase reach of intervention (intervention level)	Parent study feedback; pilot study feedback

### 3.1 Content

The primary adaptations to CARING for enCompass were focused on the content and the way content was described. First, we focused on identifying types of services that should be included as resources in eSNAP and as referral sources. Our formative work with rural caregivers and hospital staff identified several unmet social needs among rural cancer caregivers including financial needs (e.g., out‐of‐pocket medical costs, workplace inflexibility), physical needs (distance to treatment, food and housing insecurity), interpersonal needs (changing relationships, isolation), and service needs (e.g., lack of transportation, childcare, and respite; more detailed results are published elsewhere^24^). These findings resonated with prior literature, and while these were also identified needs in the primary intervention population, the scope and scale were often different in rural populations in that these barriers were more frequently identified and less easily addressed.

Many unmet needs in urban populations can be addressed by making caregivers aware of services and helping them to enroll. However, there is often limited availability and access to services in rural areas given the urbanization of many healthcare and ancillary services (e.g., therapists, home care, pharmacies). Similarly, infrastructure in rural areas is often limited such that roads can be difficult to navigate in inclement weather with few public transportation alternatives and poor or non‐existent broadband access can limit virtual options.^28^ We determined our pool of available services would likely be smaller, but that we would need to specifically address transportation and telecommunication barriers to meet the needs of a rural caregiving population. These findings reinforced that CARING's focus on developing and leveraging informal social networks along with educating caregivers about available formal services is especially important for rural caregivers.

**FIGURE 1:**
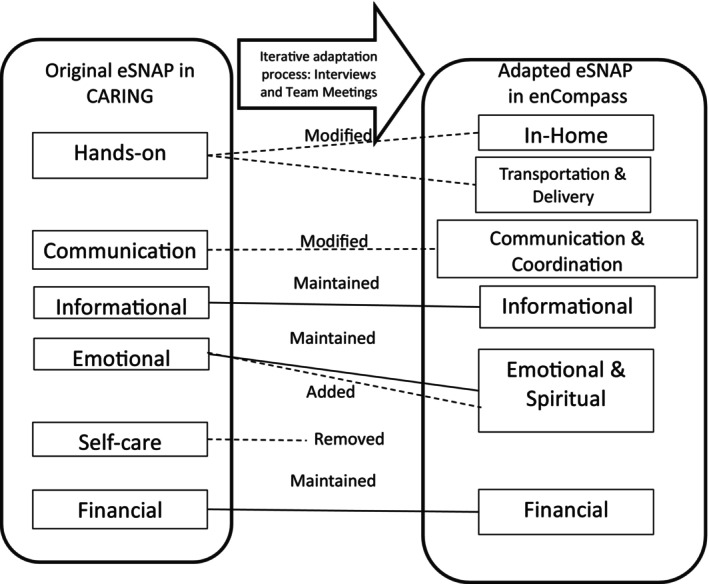
Modifications made to the electronic Social Network Assessment Map web‐based tool, from CARING to enCompass.

While interviews reinforced some existing aspects of CARING's content, there were also some changes to content. The most significant of these were changes to the social support domains listed in eSNAP to better resonate with caregiver participants (Figure 1). The original social support dimensions of eSNAP in CARING included: Hands‐on, Informational, Communication, Financial, Emotional, and Self‐care. The domains for the adaptation included In‐home, Informational, Communication, and Coordination, Financial, Emotional, and Spiritual, and Transportation and Delivery. Hands‐on was divided into In‐Home and Transportation and Delivery given that some caregivers felt that they might have a different set of people that they would feel comfortable with coming into their homes versus who they would feel comfortable with providing transportation. One caregiver reported, “people's needs could be totally different in their homes.” Another described “sometimes when people are going through this, they're private… and they don't want their family members.” Thus, we decided to separate in‐home support from transportation and delivery.

Coordination was added to Communication to signify that this set of supports could really help with logistical support as well as messaging. Some caregivers reported on these as tasks they would assign to different supporters, others saw that this type of support could come from one person, for example: “You got to have somebody at the control center delegating and coordinating and making sure it all works together.” Nearly all caregivers we spoke with mentioned reliance on places of worship and communities of faith as being helpful and supportive. For example, during our qualitative interviews, one caregiver referenced relying on “prayer warriors,” and another indicated that the category immediately made her think of “pastors at church.” We added “Spiritual” to the “Emotional” support category to signal to future participants this potential source of support, but decided to combine it with the “Emotional” support category so as not to create a category that might have resonance with some caregivers.

Finally, we removed Self‐Care as a domain as this did not resound with caregivers as a source of support, although it was deemed important as goal for caregivers to pursue during the intervention and is addressed specifically in navigation sessions. Content of the coaching sessions largely remained the same, with the exception of replacement of a close‐ended self‐reflection prompt with a more open‐ended one. In CARING, caregivers are asked to rate themselves in four emotional domains (stressed, frustrated, in control, hopeful) before and after each session to set the tone for the sessions and help participants self‐reflect, but discussion suggested this may be too limiting. As a result, enCompass coaches use a more open‐ended prompt, asking participants to provide three words at the beginning and end of each session. Participants are provided a starting list of emotional words to use as prompts, although participants were not required to go off only that list.

### 3.2 Training

Training modifications focused on reframing the interventionist's title and tailoring the training. The role of “navigator” was relabeled as “coach.” The primary reason for this was that within the institution where the intervention was being adapted, patient navigators were already well embedded in clinics and clinical stakeholders suggested a new title would reduce role confusion. The term coach was endorsed in caregiver interviews as sounding favorable yet separate from a clinician, such as a therapist. The navigator/coach training and type of personnel were also adapted. The original navigator/coach training included extensive education on neuro‐oncology and integration within that specific clinic; however, with the change of scope to include patients with multiple types of cancers, training was simplified and broadened to be more general. Qualitative interviews with hospital staff provided specific wording and approach suggestions to help introduce our study to caregivers. A member of the navigation team said, “your eyes will show how much you care… [ask] ‘is it okay if I sit down?’” These suggestions were instrumental in helping the team prepare for recruitment and intervention sessions.

We also met with the original CARING intervention team to review the navigation/coaching manual and made adaptations to the enCompass participant‐facing workbook. Mock coaching sessions were first held with an experienced supportive care interventionist, and then that interventionist trained two new coaches. Moving forward, coaching sessions are recorded for random intervention fidelity checks, updates on all current participants are discussed, and coaches send out weekly updates on all currently coaching participants. Additionally, to enhance scalability for a future larger trial and reduce costs, we are incorporating social work and clinical psychology graduate students as coaches in addition to research staff.

### 3.3 Context

While the CARING Study eSNAP tool is web‐based, which research has shown may be problematic given disparities in internet access,^29^ the app was designed for low‐bandwidth connections, and procedures have also been developed in the parent study to accommodate off‐line use. Navigation sessions are designed to be completed by phone, which is often more accessible for rural residents and those who are less tech‐savvy. As such, we determined that these features would not need to be modified at this time and that we would be able to retain the same delivery method as the parent study.

However, based on feedback from the parent study and validated through our pilot work, we made several other study‐level contextual modifications. First, we eliminated the requirement for patient participation to expand our reach. While collecting patient data is important to establish potential interdependent effects, patient consent and participation was a major limiting factor in enrollment. Our pilot study demonstrated higher enrollment rates than the parent study, confirming this decision. Similarly, based on stakeholder feedback, team discussion, and usage in our pilot study, we identified several unique types of resources that should be included in eSNAP and offered by coaches, both due to the differing nature of the disease (i.e., different cancer type support groups) as well as the unique rural context (more focus on transportation vouchers). Finally, given that we expanded the intervention to allow multiple cancer sites but limit to rural participants, we developed study recruitment and screening strategies to reflect this change. First, we screened for patient rural residence in the electronic health record by zip code to first narrow down who we would approach. This method also provided information on upcoming clinical appointments that would provide opportunity for in‐person encounters. Then, given multiple demands for time patients are faced with and anticipation of caregiver reluctance to seek support and findings from our formative work, we decided to optimize recruitment by approaching patients and caregivers during infusion appointments. This strategy allowed us to introduce the study to patients and caregivers during a time when they might be waiting for hours at a time with less likelihood for interruption and more receptivity to the intervention, as predicted by caregivers and clinicians interviewed in our formative work.

## 4 DISCUSSION

There is a clear need to better support cancer caregivers living in rural areas.^24^, ^30^, ^31^ One way to do this directly is to adapt existing efficacious interventions for rural caregivers. This paper describes our team's process in adapting the CARING intervention into enCompass for rural cancer caregivers and describing the changes made using the FRAME‐IS framework. A single‐arm feasibility trial of the adapted intervention, named enCompass Carolina, is currently ongoing. Key lessons learned in the intervention adaptation process center around intervention selection, involvement of stakeholders, and the importance of regular communication with the developers of the original CARING intervention.

First, we selected an intervention that with strong theoretical mechanisms, support‐seeking and building self efficacy, relevant to our target population, rural caregivers. Our formative work enabled us at the macro‐level to identify population patterns of rural caregivers, which included identifying that the biggest unmet needs were around accessing services,^32^ and at the local context level learn about the experiences of rural cancer caregivers directly, including both notable displays of resilience and reluctance in asking for help.^24^ The intervention we selected for adaptation, CARING, was built to address the caregiver stress process model through changing appraisal and coping mechanisms by fostering support‐seeking.^16^ Thus, the CARING intervention was a strong candidate for us to build from. Additionally, while many aspects of an intervention, such as modality, terminology, and so forth, can be modified, changing the core aspects of the intervention can change the mechanism for effect and potentially diminish its impact. Furthermore, identifying an intervention that requires less adaptation is likely to be simpler and use fewer resources. Interventions with strong theoretical foundations provide mechanistic evidence, guidance for core aspects of the intervention, and clarity regarding measurable outcomes. The CARING intervention targeted key caregiving needs—leveraging informal support and taking advantage of existing formal resources—identified as important for rural caregivers. The intervention is delivered in a way that was likely to be acceptable and feasible for participants—primarily by phone with human interaction from a coach to help guide them through study activities and help build coping strategies.

A second lesson was the critical importance of regularly engaging with stakeholders. Adaptations rely on the opinions of both the target population as well as other stakeholders. For example, simply converting an intervention to be online to reach more people may not succeed in the way that might be expected; often rural individuals have more difficulty with consistent online access, and many prefer other modalities more tailored to their specific experience.^28^ This is also seen in simple translations of interventions. Rather, researchers should focus on true cultural adaptation. In our study, this was evident in how we reframed certain aspects of the intervention based on our constituent interviews. Although the content was not significantly altered, framing of certain concepts shifted to better resonate with the target audience. For example, several participants in the adaptation interviews mentioned reliance on communities of faith, and so enCompass more explicitly refers to spiritual support. Other constituent opinions, including clinician and health system administrators, are also important; for example, the navigator and coach role are functionally the same, but changing the title reduced confusion institutionally and within the clinic, enhancing acceptability for those we hope will help scaffold the intervention to ensure its continued success.

A third lesson was the importance of regular communication in our intervention adaptation. Certainly, good communication with those in our target population and within the clinics and institutions is important to ensure buy‐in and appropriate feedback to ensure acceptability and feasibility. However, particular to the adaptation process, communication with the developers and staff of the parent intervention offered the opportunity to save an extensive amount of time through document sharing, discussing existing processes, as well as providing insight into why certain decisions were made and what aspects could be improved. However, this communication relies on a foundation of trust. Although the goal of developing scientific interventions is to help people, there are considerations around intellectual property, sharing credit, and the need for individual academic success that can sometimes limit collaboration and inhibit building on existing interventions. In our case, we approached the adaptation of CARING in a spirit of collaboration and had frequent initial and ongoing discussions about ownership, how to work together, and how to share credit. These can be uncomfortable, but openness and generosity can help advance a shared mission.

The structured and iterative process we undertook was also designed to ensure better implementation outcomes.^3^ By emphasizing caregiver and hospital staff input to our adaptation, we hope to ensure high acceptability from caregivers and increase potential adoption from healthcare administrators. By working directly with the developer of the CARING study (MR) and documenting our processes with a detailed protocol and this manuscript, we maximize feasibility and fidelity. Finally, after we examine efficacy, our documentation will aid in determining micro‐costing for future cost‐effectiveness research.

The majority of existing interventions, which have been primarily conducted in dementia caregiving, focus on self‐care and stress management rather than an explicit focus on social support.^10^ In addition, there are very few interventions tailored to the specific circumstances of rural dwelling cancer caregivers.^11^ One intervention, ENABLE, targets both rural and African–American caregivers of patients with recently diagnosed advanced cancer, is grounded in stress and coping theory, and focuses on improving caregiver skills, decision‐making, and advanced care planning. Similar to enCompass, ENABLE is designed to be delivered by telephone by lay navigators and has shown strong potential in a pilot randomized trial.^33^ One distinction of enCompass is that it focuses on leveraging social support, rather than individual coping practices; it is likely that a diversity of models are warranted to meet different families' needs. Not every intervention will easily mold to fit the rural population; it is important to be strategic with intervention adaptations in order to enhance effects without eliminating the key mechanisms.

## 5 CLINICAL IMPLICATIONS

Adapting an intervention to match with local population needs ensures greater potential fit, including relevance and sustainability, as well as greater potential engagement from clinicians. Our interviews with hospital staff yielded some signs of frustration with some existing state‐based support networks, that faster connections could be made when contacts were more personal. Our iterative process also revealed several points of tradeoff between creating standard processes versus localized adaptations. We recognize our future work will also have to balance what is core to the intervention and what can and/or must be modified to ensure the intervention fits caregiver needs, responds to clinician workflows, and can be sustained in the long term.

## 6 STUDY LIMITATIONS

We present one example of an adaptation and we conducted this with support of the original intervention development team. Limitations of our study include the use of one geographic area (rural cancer caregivers in the Southeast United States) and additional limitations inherent to qualitative research—small sample size, positionality bias of the research team, possible participant recall bias. Adaptations for less similar populations—such as those across different healthcare models—may require more work and better focus on the core mechanisms of the intervention to preserve fidelity. Developing collaborative teams across institutions may not be easily replicated, however shifting cultures to team science approaches increases the potential likelihood of collaboration. Substantial effort and institutional support are needed to ensure success. In addition, there is a need for more intentional collaborative opportunities among behavioral scientists, who have developed promising interventions with those that can facilitate further tailoring and increased reach to populations with high unmet needs.

## 7 CONCLUSIONS

We report on the process used to modify the CARING study designed for neuro‐oncology caregivers into enCompass Carolina for caregivers of rural cancer patients. We undertook a structured and iterative process to ensure that the conceptualization of social support, the framing of strategies to build social support, and the resources offered to participants would resonate with the new target population. Next steps include a randomized controlled trial to evaluate the efficacy of enCompass in a rural population across cancer sites, and then evaluation across multiple geographic sites for additional tailoring to context. Finally, investigating facilitators and barriers to implementing enCompass in rural cancer delivery settings will be critical to ensuring scalability and long‐term sustainability.

Adaptation and tailoring behavioral interventions are often appropriate strategies to consider for resource limited settings, including supportive care delivery. Behavioral intervention trialists should continue to refine adaptation processes to increase efficiency while preserving resonance for target populations.

## AUTHOR CONTRIBUTIONS

**Erin E. Kent:** Conceptualization (lead); data curation (lead); formal analysis (lead); funding acquisition (lead); investigation (lead); methodology (lead); project administration (lead); resources (lead); software (lead); supervision (lead); validation (equal); visualization (equal); writing – original draft (lead). **Kelly R. Tan:** Conceptualization (supporting); methodology (supporting); writing – original draft (supporting). **Zev M. Nakamura:** Conceptualization (supporting); investigation (supporting); methodology (supporting); resources (supporting); visualization (supporting); writing – original draft (supporting). **Jesse Kovacs:** Project administration (supporting); resources (supporting); software (supporting); supervision (supporting); writing – original draft (supporting). **Mindy Gellin:** Project administration (supporting); resources (supporting); software (supporting); writing – original draft (supporting). **Allison Deal:** Conceptualization (supporting); formal analysis (supporting); software (supporting); writing – original draft (supporting). **Eliza M. Park:** Conceptualization (equal); data curation (equal); formal analysis (equal); funding acquisition (equal); investigation (equal); methodology (equal); project administration (equal); resources (equal); software (equal); supervision (equal); writing – original draft (supporting). **Maija Reblin:** Conceptualization (equal); writing – original draft (equal).

## FUNDING INFORMATION

Funding for this work was provided by a grant from The Duke Endowment and funds from the University Cancer Research Fund.

## CONFLICT OF INTEREST STATEMENT

The authors have no conflicts of interest to declare.

## Data Availability

Data available on request from the authors.
